# The Open Access Advantage

**DOI:** 10.2196/jmir.8.2.e8

**Published:** 2006-05-15

**Authors:** Gunther Eysenbach

**Affiliations:** ^1^Centre for Global eHealth InnovationTorontoCanada

**Keywords:** Knowledge translation, open access, bibliometrics, open source

## Abstract

A study published today in PLoS Biology provides robust evidence that open-access articles are more immediately recognized and cited than non-OA articles. This editorial provides some additional follow up data from the most recent analysis of the same cohort in April 2006, 17 to 21 months after publication. These data suggest that the citation gap between open access and non-open access papers continues to widen. I conclude with the observation that the “open access advantage” has at least three components: (1) a citation count advantage (as a metric for knowledge uptake within the scientific community), (2) an end user uptake advantage, and (3) a cross-discipline fertilization advantage. More research is needed, and JMIR is inviting research on all aspects of open access. As the advantages for publishing open access from a researchers' point of view become increasingly clear, questions around the sustainability of open access journals remain. This journal is a living example that "lean									publishing" models can create successful open access journals. Open source tools which have been developed by the Public Knowledge Project at the University of British Columbia with contributions from the Epublishing & Open Access group at the Centre for Global eHealth Innovation in Toronto are an alternative to hosting journals on commercial open access publisher sites.

## Citation Advantage of Open Access Articles

*PLoS Biology* today publishes a study authored by JMIR founding editor and publisher Gunther Eysenbach on the impact of publishing papers as open access articles, concluding that open access articles have a clear citation advantage over non–open access articles (see [1] and Multimedia Appendix 1).

The study, already referred to as a landmark study by colleagues, is the first publication providing robust evidence for a citation advantage of articles published “originally” as open access articles (so-called “gold road” to open access) compared with articles published in the same journal as non-immediate open access articles. This kind of comparison became possible because the journal *PNAS* *(Proceedings of the National Academy of Sciences),* under the visionary leadership of the late Nicholas Cozzarelli, started an experiment in mid-2004 offering authors the option of paying an additional fee to make their article freely available immediately after publication. *PNAS* became one of the first “hybrid” journals. The resulting mix of open access and non–open access articles published in *PNAS* represents an ideal study cohort. The study published today in *PLoS* [1] is the first of a series of papers that will follow up this cohort over several years, with today’s paper describing the citation behavior over the early period of up to 16 months after publication, collecting citation data every 6 months.

**Figure 1:**
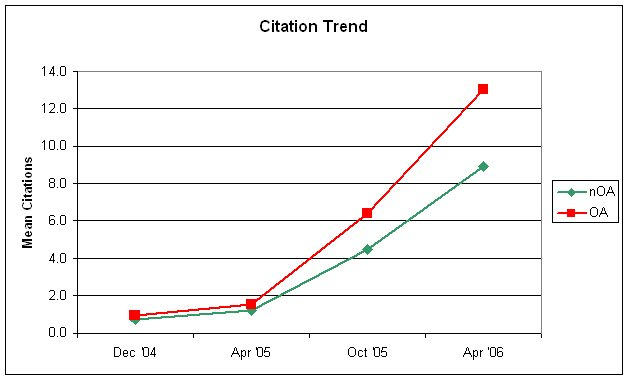
Citation trend in terms of mean number of citations at different points in time (for PNAS publications published in the second half of 2004)

Figure 1 and Table 1 are updated versions of the figures presented in the *PLoS Biology* article, with the most recent study point of April 2006 being added (representing a follow-up time of up to 21 months after publication). It shows the (unadjusted) citation advantage of open access articles over non–open access articles, with the gap continuing to widen. This citation advantage remains significant even when adjusted in multivariate regression models to correct for differences in article and author characteristics (not shown here, see [1] and Multimedia Appendix 1 for details).

**Table 1:** Updated version of Table 2 in the Eysenbach study [1], with the most recent study point April 2006 added, showing unadjusted citation rates of PNAS articles published in the second half of 2004

	**Non–Open Access** **(n = 1280)**	**Open Access** **(n = 212)**	RR^*^ (95% CI)	***P* value**
**Uncited Articles**				
December 2004 (%)	1056 (82.5)	170 (80.2)	1.0 (1.0-1.1)	*P* = .44^†^
April 2005 (%)	627 (49.0)	78 (36.8)	1.3 (1.1-1.6)	*P* = .001^†^
October 2005 (%)	172 (13.6)	11 (5.2)	2.6 (1.4-4.7)	*P* < .001^†^
April 2006 (%)	70 (5.5)	3 (1.42)	3.9 (1.2-12.2)	*P* = .009
				
			**% Difference**	
**Mean Number of Citations**				
December 2004 [median] (SD)	0.7 [0] (2.0)	0.9 [0] (2.8)	29	*P* = .35^‡^
April 2005 [median] (SD)	1.2 [1] (2.0)	1.5 [1] (2.5)	25	*P* = .002^‡^
October 2005 [median] (SD)	4.5 [3] (4.9)	6.4 [4] (10.4)	42	*P* < .001^‡^
April 2006 [median] (SD)	8.9 [7] (8.5)	13.1 [9] (20.4)	47	*P* < .001

^*^RR = relative risk for non–open access articles not being cited by the time of analysis

^†^Comparing the proportion of uncited articles in the open access group with the proportion of uncited articles in the non–open access group (Fisher’s exact test)

^‡^Comparing the (ranked) number of citations between the groups (Wilcoxon rank test)

Claims of an “open access impact advantage” may sound familiar, as open access “archivangelists” have talked about such an advantage for years [2]; however, this previous evidence is scientifically weak [1,3], comes primarily from the “self-archiving” (“green road”) variant of open access articles, and has failed to convince open access critics such as Jeffrey Aronson, chairman of the editorial board of the subscription journal *British Journal of Clinical Pharmacology*, who wrote in the *BMJ* that “there is no evidence that this [increasing citations] will happen” [4]. Indeed, previous studies were mostly cross-sectional and largely ignored possible confounders (ie, differences in other characteristics between open access and non–open access articles that may be independently responsible for citation differences). These previous studies culminated in less than credible, sweeping conclusions such as “open access increases the impact of articles in [subject x] by x%.” By stratifying their observations by subjects, the authors of such statements implicitly acknowledged that the subject is an important confounder, but they failed to consider other confounders, such as the number of authors, which may be independent predictors for citation counts and which may differ between the groups. Multivariate analysis allows for control of these factors, that is, determining the influence of open access status if all these other factors are held constant.

The other aspect that has been previously ignored is the time factor (ie, time after publication) as a covariate that determines the actual strength of the citation advantage. It is unrealistic to assume that the open access advantage, as measured as the rate ratio of new citations per time period (per year or per month), is the same 1 year, 3 years, 5 years, 20, or 100 years after publication. Rather, what can be expected is that, after a sharp increase of the open access advantage shortly after publication, over time, the citation advantage is likely to diminish. Figure 1 shows that the rate of new citations (the steepness of the slope) is still larger in the open access group, even in the April '06 analysis, 17 to 21 months after publication. However, ultimately both lines will become parallel, indicating an equal citation rate in both groups, as *PNAS* articles in the nOA group are now also freely accessible (note that one can not expect the citation rates to become equal immediately after 6 months, when articles from both groups are free, as it often takes months or years before a manuscript gets published and the bibliography of that published manuscript shows up in the ISI database. Hence, the effect of authors citing preferentially an open access article in late 2004/early 2005 can still be observed today).

The cohort study published today [1] provides robust evidence showing the independent effect of publishing an article in an open access journal, while allowing us to track the citation behavior over a number of years after publication. As discussed in the article [1] and the accompanying editorial [3], the observed citation advantage has significant policy implications, but bibliometrics (counting citations) only tells one part of the story and is only one component of the construct we call open access advantage.

## Beyond Citations

**Figure 2:**
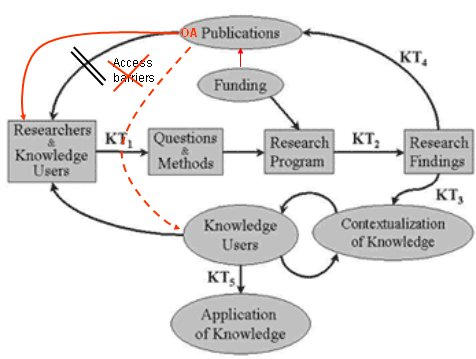
The Knowledge Translation Cycle (Source: Canadian Institutes of Health Research), illustrating (in red) the impact of open access.

The traditional knowledge translation cycle (Figure 2) actually consists of two separate cycles: (1) the translation process (in the upper part of the figure) that takes place within the scientific community, mainly through scientific publications, and (2) the translation process of research to the end-user (in the lower part of the figure) that is facilitated by other mechanisms. This diagram illustrates the implicit assumption that, traditionally, knowledge users who are not researchers (policy makers, consumers, journalists) do not necessarily read scientific publications. In our 7 years of experience with this journal (*JMIR*), we have received many anecdotal reports from authors and research users testifying that open access publication can help to bridge this gap. Policy makers and end-users are much more likely to “google” for evidence than to do a formal literature search [5,6], and even if they come across a subscription-based scientific paper through Google, they are unlikely to actually order it. Only if a publication is open access will end-users skim and eventually read it, or contact the author, after they discovered that it is relevant to the policy (or practical) question at hand. We know that *JMIR* is used as much by patients and other nonresearchers (eg, policy makers) as it is by eHealth researchers, and we know from our authors that they are often contacted by “atypical” readers (knowledge end-users) who bumped into their article by pure chance, which they would never have done had the article been published in a subscription-based scholarly journal.

Another aspect of the open access advantage is that open access may increase the chance for what I call “cross-discipline fertilization” within the scientific community. I first made this observation when analyzing the journals in which *JMIR* articles are cited. Other than traditional subscription-based journals from the health informatics field, *JMIR* articles are more likely to be cited in general medical journals or specialist medical journals (ie, articles are not only cited within the medical informatics community). In contrast, articles in traditional medical informatics journals tend to be cited mainly in other medical informatics journals, rarely crossing the boundaries of their narrow discipline. While this may also have to do with the broader scope of *JMIR,* this observation was an early indicator for the open access cross-discipline fertilization advantage. Preliminary (yet unpublished) analysis of cited articles from the *PNAS* cohort seems to corroborate this observation.

In summary, I conclude that the open access advantage really has at least three components: (1) a citation count advantage (as a metric for knowledge uptake within the scientific community), (2) an end user uptake advantage, and (3) a cross-discipline fertilization advantage. In the case of preprints and self-archiving, one may add a quality advantage to this list, as prepublication discussion of articles may lead to quality improvements [7,8]. All of these advantages are of course the result of greater visibility within and beyond the scientific community.

Note that this view differs from how previous researchers have characterized the open access impact advantage in the context of self-archiving [2]. The *PNAS* cohort confirms the citation count advantage; however, the other aspects of the open access advantage are more difficult to measure, and further research into the more qualitative advantages of publishing in an open access journal, namely cross-discipline fertilization and uptake by end-users, is needed.

## More Research Needed: A Call for Papers

It is clear that much more rigorous research is required in this field. One question that arises for researchers is where to publish this kind of research. Traditional scientometrics and information science journals are all subscription based and only read by a few specialist researchers. A suitable publication outlet for this kind of research should of course be open access.

*PLoS Biology* has made it clear in their editorial that it does not intend to make *PLoS* a home for bibliometric studies [3]—even if they are about open access. So where should researchers send their best research on open access? We would like to offer *JMIR* as a peer-reviewed outlet for such research, even if it transcends the health sector. After all, the original mission of *JMIR* was to publish research on the impact of the Internet on medical and scientific communication and information. Open access would not be possible without the Internet. Thus, we are very interested in receiving submissions (in particular, those with original data) on the effects and ramifications of open access, and the many aspects that surround this issue.

## Sustainability

As the advantages for publishing open access from a researchers' point of view become increasingly clear, questions around the sustainability of open access journals remain. Open access giants such as PLoS or Biomed Central are often mentioned as *the* representatives of the open access publishing movement, and it is quickly pointed out that the way they operate is not sustainable. What is often forgotten is that these publishers are not the only open access publishers (they were not even the first open access publishers - with publishers like *BMJ*, *Medscape*, or *JMIR* being the true pioneers), and they are certainly not typical representatives. The majority of open access journals operate using a lean publishing model, and many of them are financially sustainable. This journal is a living example that lean publishing models can create successful open access journals. In the light of growing concern and disgruntlement among editors with commercial open access giants such as BioMed Central [9], we wish to remind researchers that open source tools for publishing open access journals are readily available and have become increasingly sophisticated. The Epublishing & Open Access group at the Centre for Global eHealth Innovation, under the technical leadership of MJ Suhonos and scientific direction of Gunther Eysenbach, has not only been a user, but also a major contributor to open source tools such as Open Journal Systems originally developed by the Public Knowledge Project [10]. Bringing these tools up to speed in terms of XML publishing compatible with the NLM-DTD has been a major contribution of the group, which not only publishes *JMIR,* but also donates tools, technology, software, and experience to the scientific community, that is, to anyone who wants to create a new open access journal (see http://www.jmir.org/?Start_a_new_journal for details). While we are convinced that open access is the future, and with all of our sympathies for PLoS and BMC, we also hope that the future of open access does not solely rely on a quasi-monopoly of only two large open access publishers.

## Multimedia Appendix 1

Full text of the Eysenbach study [1]

## Multimedia Appendix 2

Full text of the accompanying editorial [3]

## Multimedia Appendix 3

PLoS Press Release
