# Supplementary material for: The Open Access Advantage
Source: J Med Internet Res. 2006 May 15;8(2):e8. doi: 10.2196/jmir.8.2.e8 (PMC1550699; doi:10.2196/jmir.8.2.e8)
Supplement: Supplementary file 3 [file jmir_v8i2e8_app3.pdf]

Embargoed for release: 15-May-2006 20:00 Eastern US Time

Contact: Paul Ocampo
press@plos.org
415-624-1224
Public Library of Science

## Free access to science speeds its use

Most of the science published today is in journals that can only be read by subscribers. The Public Library of Science (PLoS) is part of a movement advocating the unrestricted dissemination of scientific information: open-access (OA) publishing. In this issue of the open-access journal PLoS Biology, Gunther Eysenbach provides robust evidence that open-access articles (OA articles) are more immediately recognized and cited than non-OA articles. As such, it adds objective support to the belief that open-access publication speeds up scientific dialog between researchers and, consequently, should be extended to the whole scientific literature as quickly as possible.

Since most open-access journals are new, comparisons of the effects of open access with established subscription-based journals are easily confounded by age and reputation. In the current study, Eysenbach compared citations compiled by Thomson Scientific (formerly Thomson ISI) to individual articles published between June 2004 and December 2004 in the same journal--namely, Proceedings of the National Academy of Sciences (PNAS), which announced its open-access option for authors on June 8 of that year, with an associated publication charge of US$1,000. Non-OA articles in PNAS are subject to a six-month delay before the published article becomes publicly available.

The results of this natural experiment are clear: in the 4 to 16 months following publication, OA articles gained a significant citation advantage over non-OA articles during the same period. They are twice as likely to be cited 4 to 10 months after publication and almost three times as likely between 10 and 16 months. Given that PNAS delays open access for only six months, the disparity between OA and non-OA articles in journals where the delay is longer or where articles are available to subscribers only is likely to be even greater.

PNAS was one of the first journals to offer an open-access option to its authors. However, such hybrid journals are increasing: Blackwell, Springer, and Oxford University Press now provide this option as well. This means that similar experiments can be replicated. Moreover, although the evidence from the current analysis argues most strongly for a time advantage in citation for OA articles, a study over longer periods will reveal whether this translates into a sustained increase in the number of citations. In the meantime, open-access advocates should be emboldened by tangible evidence for what has seemed obvious all along.

Of course, PLoS Biology has a strong and vested interest in publishing results that so obviously endorse our existence. Moreover, the author of the article is also an editor of an open-access journal. In an accompanying editorial, the editors of PLoS Biology discuss the careful evaluation process and peer review of this paper.

###

**Citation: Eysenbach G (2006) Citation advantage of open access articles. PLoS Biol 4(5): e157.**

**PLEASE ADD THE LINK TO THE PUBLISHED ARTICLE IN ONLINE VERSIONS OF YOUR REPORT: http://dx.doi.org/10.1371/journal.pbio.0040157**

PRESS ONLY PREVIEW OF THE ARTICLE: http://www.plos.org/press/plbi-04-05-eysenbach.pdf

Related PLoS Biology Editorial article:

Citation: MacCallum CJ, Parthasarathy H (2006) Open access increases citation rate. PLoS Biol 4(5): e176.

PLEASE ADD THE LINK TO THE PUBLISHED ARTICLE IN ONLINE VERSIONS OF YOUR REPORT: http://dx.doi.org/10.1371/journal.pbio.0040176

PRESS ONLY PREVIEW OF THE ARTICLE: http://www.plos.org/press/plbi-04-05-editorial.pdf

CONTACTS: Gunther Eysenbach, MD MPH
University Health Network
Centre for Global eHealth Innovation
190 Elizabeth St
Toronto, ON Canada M5G2C4
+1-416-786-6970
+1-416-340-3595 (fax)
geysenba@uhnres.utoronto.ca

Hemai Parthasarathy, PhD
Managing Editor, PLoS Biology
Public Library of Science
185 Berry Street, Suite 3100
San Francisco, CA 94107 USA
+1-415-624-1205
+1-415-546-4090 (fax)
hemai@plos.org

Mark Patterson, PhD
Director of Publishing
Public Library of Science
7 Portugal Place
Cambridge CB5 8AF UK
+44-(0)1223-463-346
mpatterson@plos.org

PLEASE MENTION THE OPEN-ACCESS JOURNAL PLoS BIOLOGY (www.plosbiology.org) AS THE SOURCE FOR THESE ARTICLES AND PROVIDE A LINK TO THE FREELY-AVAILABLE TEXT. THANK YOU.

All works published in PLoS Biology are open access. Everything is immediately available--to read, download, redistribute, include in databases, and otherwise use--without cost to anyone, anywhere, subject only to the condition that the original authorship and source are properly attributed. Copyright is retained by the authors. The Public Library of Science uses the Creative Commons Attribution License.

For news media only:
PRESS ONLY PREVIEW OF THE ARTICLE: http://www.plos.org/press/plbi-04-05-eysenbach.pdf
